# Preparation and consideration for establishment of an isolation maternity unit in a tertiary hospital during COVID-19 pandemic

**DOI:** 10.1186/s12884-022-04643-w

**Published:** 2022-04-13

**Authors:** Yingke He, Yvonne Wan Yu Wong, Alvin Jia Hao Ngeow, Eileen Yilin Sim, Benjamin Pei Zhi Cherng, Sridhar Arunachalam, Selina Kah Ying Ho, Wei Ching Tan, Un Sam Mok

**Affiliations:** 1 Department of Anaesthesia, Division of Anesthesiology and Perioperative Medicine, Singapore General Hospital, Outram Road, Singapore, 169608 Singapore; 2 Department of Obstetrics and Gynaecology, Division of Surgery and Surgical Oncology, Singapore General Hospital, Outram Road, Singapore, 169608 Singapore; 3 Department of Neonatal and Developmental Medicine, Division of Medicine, Singapore General Hospital, Outram Road, Singapore, 169608 Singapore; 4 Department of Infectious Disease, Division of Medicine, Singapore General Hospital, Outram Road, Singapore, 169608 Singapore

## Abstract

The SARS-CoV-2 pandemic is rapidly evolving and remains a major health challenge worldwide. With an increase in pregnant women with COVID-19 infection, we recognized an urgent need to set up a multidisciplinary taskforce to provide safe and holistic care for this group of women. In this review of practice in a tertiary hospital in Singapore, we discuss the key considerations in setting up an isolation maternity unit and our strategies for peripartum and postpartum care. Through teleconsultation, we involve these women and their families in the discussion of timing and mode of birth, disposition of babies after birth and safety of breastfeeding to enable them to make informed decisions and individualize their care.

## Introduction

Severe Acute Respiratory Syndrome Coronavirus 2 (SARS-CoV-2) is a novel coronavirus that has developed into a world pandemic. SARS-CoV-2 related disease (COVID-19) has also caused significant mortality and morbidity worldwide since December 2019, with the total global death toll exceeding five million as of Jan 2022. Governments and the World Health Organization (WHO) have made tremendous efforts to increase global vaccination rate. As the virus continues to evolve, new variants have emerged during the course of the pandemic causing a rapid emergence within populations due to an increase in transmissibility [1].

COVID-19 in pregnancy presents a unique challenge due to limited data on management strategies and its association with more severe disease in pregnant women. CDC surveillance data on outcomes in approximately 400,000 reproductive-aged women with symptomatic, laboratory-confirmed COVID-19 found that pregnant women had significantly higher rates of intensive care unit (ICU) admission, mechanical ventilation, extracorporeal membrane oxygenation and death [2]. These poorer outcomes may be attributed to the physiological and immunological changes during pregnancy, such as reduced functional residual capacity of lung, elevation of the diaphragm, increased metabolic rate and altered cell immunity. There is also an increased incidence of poorer obstetric outcomes, such as preterm deliveries, fetal distress and caesarean deliveries in this group of women. Vaccination has been shown to be effective in preventing severe illness, hospitalization and death from COVID-19 in the general population with no known harmful effects to the fetus. However, some pregnant women have reservations about receiving vaccination in pregnancy due to its relative novelty and limited data on long-term effects on the fetus [3–5].

The earliest experience of COVID-19 related peripartum care came from China, where the disease first emerged. These pregnant women almost exclusively gave birth via caesarean section and were separated from their babies for a minimum duration of 2 weeks with no breastfeeding [6]. The recent Royal College of Obstetricians and Gynaecologists (RCOG) guidelines as well as other literature have suggested that normal vaginal births are considered safe as the risk of vertical transmission has been proven to be low [7, 8]. Similarly, breastfeeding and rooming-in, the practice of placing the infant in the same room as the mother for 24 h a day after delivery, may also be possible if infection control measures have been strictly observed [9, 10]. However, limited literature is available on the practicality of vaginal birth in COVID positive women and continuation of postnatal bonding with the neonate.

In this paper, we will share our experience of setting up a maternal-fetal unit in the isolation ward during the COVID pandemic at Singapore General Hospital (SGH), the largest tertiary hospital in Southeast Asia. We would also like to discuss the practical aspects of coordinating multidisciplinary specialist care for covid positive women, safe provision of care for birth in an isolation room or in the operating theatre, and anaesthesia and analgesia options during labour. Postnatal care options such as rooming-in and breastfeeding in an isolation unit are also discussed in detail.

### Multidisciplinary preparation of peripartum care in the isolation ward

Since August 2021, when the target of 80% of the Singapore population to be vaccinated against COVID-19 was attained, the Singapore government pushed ahead with a strategy of “living with COVID” as an endemic disease with gradual relaxation of restrictions [10]. Previous lockdowns and border closures were eased, introduction of self-isolation at homes for contacts of covid-19 and home recovery as the default for healthy low-risk patients with covid-19 were all part of “living with COVID” strategy [9]. Consequently, there was a tremendous uptick in daily new covid cases commencing in Sept 2021, and a concomitant rise in the number of pregnant women infected with COVID-19. Initially, most pregnant COVID-19 women were hospitalized, even those who were asymptomatic or had mild symptoms. Policies were soon revised to reserve admission for women at greater risk of severe disease, such as women with pregnancies ≥24 weeks gestation, or who were symptomatic. Conversely, women with pregnancies < 24 weeks gestation and who were asymptomatic were observed in community care facilities or isolated at home. A multidisciplinary team involving obstetricians, neonatologists, anaesthetists, infectious disease physicians, operating theatre nurses and midwives was established to provide safe and holistic care for these hospitalized women. As the team is large, close and frequent coordination of care presents additional logistical challenges on top of infection control for this group of women.

#### Effective communication and coordination of care

Multidisciplinary specialist input is of paramount importance in the care of such women. To effectively engage members from the different healthcare teams, a communication group was set up using the institution’s communication application software (Tiger connect), so that all new admissions, and any urgent new development in an existing patient’s condition may be rapidly broadcasted to all members.

In our institution, pregnant women with COVID-19 infection are admitted to the isolation ward and are primarily cared for by the infectious disease specialists. The rest of the multidisciplinary team are alerted about their admission, and will review the patient when activated by the primary physician. These women undergo a thorough assessment encompassing history taking, physical examination, baseline investigations including cycle threshold levels of COVID-19 PCR test as an estimate of transmission potential. These women are co-managed by the obstetrics team who will assess maternal and fetal well-being and monitor for signs and symptoms of labour. The frequency and mode of fetal monitoring is individualized, depending on the gestational age of the fetus, risks factors in pregnancy and existing maternal morbidities. Women who are largely asymptomatic and not in labour will have their fetuses monitored daily with fetal Doppler and those with potential maternal or fetal compromise will be monitored with a daily fetal cardiotocography. Regular updates are provided in the group by the managing obstetrician and infectious disease specialists on the woman’s health status as well as changes in birth plans.

Pregnant women with COVID-19 complications (e.g. pneumonia, respiratory distress) and those who are term are also referred to the neonatology and anaesthesia teams for further counselling. After the initial assessment by the multidisciplinary team, a teleconsultation session is conducted between the various disciplines and the couple to formulate a management plan including the need for treatment with corticosteroids for fetal lung maturation, magnesium sulphate for fetal neuroprotection and medications for COVID infection like antivirals and steroids. Issues pertinent to the peripartum and postpartum phase including mode and timing of birth, availability of various intrapartum analgesia and anaesthesia options as well as relevant baby care issues (such as breastfeeding, rooming-in options) are discussed and documented. Efforts are made to respect the couple’s wishes to facilitate bonding between mother and infant, by allowing rooming-in wherever logistically and medically feasible.

#### Transforming the isolation ward into a comfortable and safe birthing environment

**Fig. 1:**
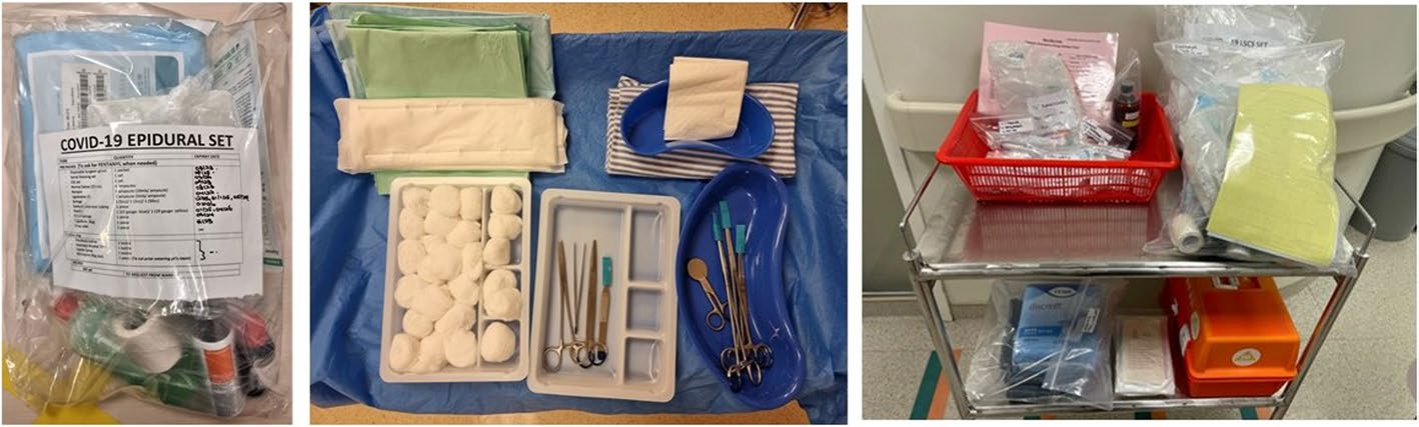
Delivery trolley containing (from left to right) the disposable epidural set, vaginal delivery set, perimortem caesarean section set and medications needed for labour and obstetric emergencies

Hospitalized COVID-19 pregnant women are not nursed in our institution’s labour ward, as there are no pre-existing single negative pressure rooms to house these women to prevent transmission of virus [11]. Thus, these women have to be admitted to single rooms within the hospital’s isolation wards which are located in a different block and level from the labour wards. Challenges were faced to transform the isolation rooms into a comfortable and safe birthing environment. Existing beds had to be exchanged for obstetric beds with a removable foot section, complete with stirrups and handles. Medical equipment needed for labour and neonatal resuscitation were stocked and kept ready in the isolation ward. A cardiotocography (CTG) machine and delivery trolley for intrapartum care and labour were prepared and parked in the isolation ward at all times. Common medications for management of obstetric emergencies such as postpartum haemorrhage and severe pre-eclampsia/eclampsia were also prepared in quick-grab ‘kits’ for individual woman (Fig. 1). The designated midwives caring for the pregnant COVID-19 women ensured that the kits were replenished immediately after each use. Portable Entonox cylinders were sourced from external vendors and made available in the birthing rooms. Midwives were taught to check the cylinders after use and replenish them as required. Portable light stands were also placed in the room to ensure good lighting for delivery and perineal repairs. Other relevant anaesthesia and neonatal equipment are also summarized in Table 1.

**Table 1:** Obstetric, anaesthesia and neonatal equipment set up in isolation ward to prepare for deliveries

**Obstetric equipment**	Cardiotocography (CTG) machine
	Disposable delivery set ➢ Disposable vaginal delivery set ➢ OmniCup and Neville Barnes forceps for assisted vaginal delivery
	Perimortem caesarean section set
	Medications for labour and obstetric emergencies ➢ Medications needed for different stages of labour ➢ Postpartum hemorrhage, pre-eclampsia/eclampsia ➢ Other resuscitation drugs
**Anaesthesia equipment**	Entonox use in isolation ward ➢ Portable entonox cylinder ➢ Tubing with demand valve and facemask
	Epidural analgesia in isolation ward ➢ Disposable epidural set ➢ Patient controlled epidural analgesia (PCEA) pump
**Neonatal equipment**	Airway and Resuscitation ➢ Transport incubator and Open care system with overhead warmer and T-piece resuscitator/disposable bag and mask kit ➢ Oxygen tank, medical air tank, high-efficiency particulate absorbing (HEPA) filter ➢ Suction machine and catheters ➢ Endotracheal tube, orogastric tube, umbilical catheter ➢ Standard neonatal resuscitation drugs
	Warming equipment ➢ Stockinet cap, towels, plastic bag ➢ Exothermic mattress

#### Creation of vaginal birth and caesarean section workflow and conduct of simulation exercise

To minimize variations in care, workflows for vaginal birth and caesarean section for COVID positive women were developed based on input from members of the multidisciplinary care team. These workflows were derived from the latest position statements by various international bodies and adapted to suit our institutional practice. To familiarize all members of the large multidisciplinary team on the ground with the workflows, simulation exercises were conducted to identify gaps for improvement. These workflows were also updated regularly to keep them current with the latest practice guidelines. With each significant modification to the workflow, another round of simulation session was organized to familiarize all key stakeholders with the changes.

### Considerations in the peripartum care of COVID positive woman

#### Maternity Service in Singapore

In Singapore, all pregnant women are seen by obstetricians at their booking visit. Most women attend the outpatient obstetric clinic every four-weekly and weekly from 36 weeks gestation for routine care. Some hospitals in Singapore have midwifery-led clinics where low-risk pregnancy women attend outpatient follow-ups from the third trimester of their pregnancy. Our labour wards are jointly managed by the obstetricians and midwives and there are no stand-alone midwifery-led units. The initial assessment on admission to labour ward and four-hourly labour ward rounds are conducted by the obstetrics team. Women with low-risk pregnancies are delivered by the midwives and those with high-risk pregnancies (such as those with macrosomic fetus, labour after previous caesarean section, previous obstetric anal sphincter injuries etc) are delivered by the obstetricians.

#### Discussing the timing and mode of birth in COVID-19 women

According to the data from the UK Obstetric Surveillance System (UKOSS) report, there is no evidence to favour one mode of birth over another in pregnant women with COVID-19 infection. A systematic review by *Walker et.al* concluded that the rate of vertical transmission of COVID-19 infection to neonates is no greater when babies are born vaginally, breastfed or stayed with their mothers after birth.

Putting together an individual’s pregnancy risk factors, previous obstetric and surgical history, severity of COVID-19 infection, we assist the woman in deciding her preferred mode of birth. All pregnant women admitted to hospital will receive Thrombo-Embolus Deterrent (TED) stockings and are offered prophylactic low molecular weight heparin (LMWH) to reduce the thrombotic risk from pregnancy and COVID infection unless labour is imminent or there is a risk of haemorrhage. A planned induction of labour at 39 weeks of gestation is recommended, which allows us to be able to omit prophylactic LMWH prior to induction/labour, arrange timing of regional anaesthesia, and ensure the availability of isolation birthing rooms and dedicated operating theatres as well as adequate staffing.

#### Conduct of normal vaginal birth in isolation ward

**Fig. 2:**
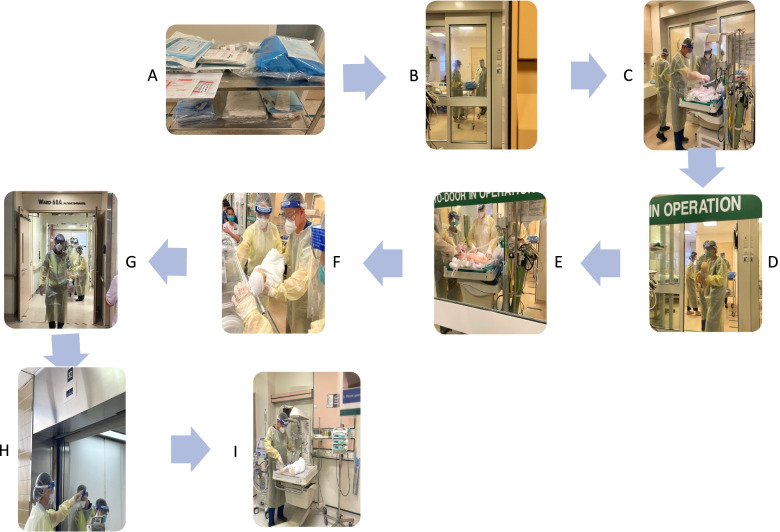
Workflow for vaginal delivery in isolation ward. **A**. Preparation of equipment and medication needed for delivery, **B**. Obstetrician and midwife delivered the baby in Negative Pressure (NEP) room, **C**. Neonatologists and staff nurse on standby to resuscitate baby as needed, **D**. Midwife hands baby over to neonatologists, **E**. Resuscitative measures carried out on baby as needed, **F**. Transfer of baby to transport incubator, **G**. Security escort during transfer of baby to Neonatal Isolation NEP room, **H**. Use of dedicated lift during transfer, **I**. Transfer of baby from transport incubator to Neonatal Isolation NEP room

Healthcare workers wear full personal protective equipment (disposable gown, gloves, face shield/goggles, N95 mask) when caring for women with COVID infection during labour. A dedicated midwife is assigned to care for woman to prevent cross-infecting other non-covid women in labour, as well as for safety, as the COVID labour room is apart from the main labour ward. When not inside the COVID labour room, the midwife may visually monitor the patient from the anteroom of the isolation room, and record hourly maternal observations such as heart rate, blood pressure and oxygen saturations on the modified early obstetric warning score (MEOWS) chart. Oxygen therapy is titrated to keep oxygen saturation levels above 94%. In view of the association of increased risk of fetal compromise during active labour in women with symptomatic COVID-19 infection, continuous external fetal monitoring is recommended and performed in our institution. These women undergo a routine four-hourly vaginal examination performed by the obstetric registrar on duty and an individualized assessment will be made regarding shortening of the second stage of labour with assisted vaginal birth in a symptomatic woman who is hypoxic or exhausted. We recommend and support the practice of delayed cord clamping and active third stage of labour. There is no evidence that the practice of delayed cord clamping and skin-to-skin contact increases the transmission of COVID-19 infection to the neonate (Fig. 2).

Upon diagnosis of the onset of labour in a covid positive woman, an update would be sent out in the secure messaging system, so that an operating theatre room that is equipped with negative pressure isolation can be set aside on standby. If there is any indication of persistent fetal distress, the obstetricians often recommend early intervention and early conversion to caesarean section, to avoid a scenario where a Category 1 caesarean section (delivery within 30 min of making the decision) is needed, as the urgency of the situation may lead to a compromise in the infection control measures, and the delay in transfer may be detrimental to the fetus [12].

#### Conduct of caesarean delivery for COVID positive women

When a COVID-19 pregnant woman requires emergency caesarean section for slow progress in labour or for other maternal/ fetal indications, the multidisciplinary team is activated early. A team brief involving the medical team of various specialties and nursing team is conducted prior to the arrival of the woman. During the team brief, roles are assigned, all patient-related concerns are discussed and instruments needed for the surgery and anaesthesia are identified.

**Fig. 3:**
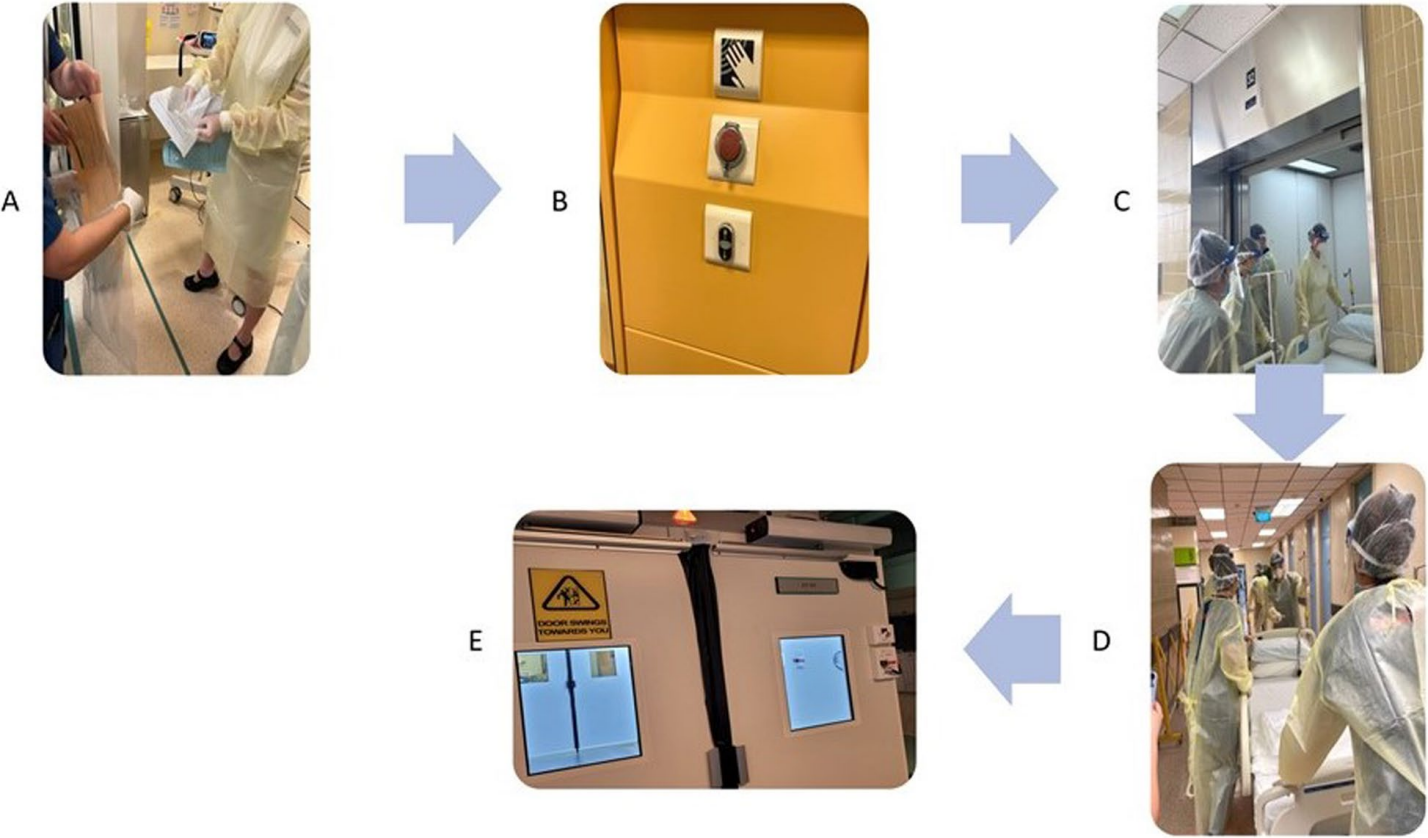
Workflow for Caesarean section delivery for isolation ward. **A**. Aseptic technique of obtaining patient consent, **B**. Emergency button to open both doors in Anteroom for emergency transfer of COVID-19 pregnant womena from NEP room to Operating room, **C**. Use of dedicated lift for transfer of mother, **D**. Security-led transfer, **E**. Negative pressure anteroom leading to Operating room

In our institution, the isolation ward is located in a separate block from the operating theatre, but are connected by sheltered walkways. Current infection control measures such as the need to secure off the corridors and lifts can cause delays during the transfer of a woman from the isolation ward to the operating theature. To minimise the delay and minimize exposure to other personnels, the security team is activated to travel ahead of the woman and her accompanying healthcare personnel. Once the caesarean section has been completed, two separate security teams are activated- one to escort the transfer of baby from the operating theatre to the Neonatal Isolation NEP room and another to escort the mother back to the isolation ward (Fig. 3).

#### Management of clinical deterioration in COVID-19 pregnant women

In our institution, pregnant women with COVID-19 infection who deteriorate have investigations performed as for non-pregnant women. These are not delayed because of concerns of potential radiation exposure to the mother and fetus. We recognize that maternal health is paramount and stabilization of the woman’s condition takes precedence. The care of these women is escalated with low threshold for ICU (intensive care unit) admission due to an increased risk of COVID related morbidity and rapid respiratory deterioration. Oxygen therapy is titrated to achieve target saturations of 94–98%. Use of prone positioning and high flow nasal oxygen therapy for women with moderate to severe acute respiratory distress syndrome (ARDS) has also been shown to improve respiratory function and avoid need for intubation.

After the initial assessment and stabilization of the woman, we will arrange for an urgent multidisciplinary team meeting to discuss the medical care of the woman and commencement of medical therapy according to results of the RECOVERY trial (corticosteroid therapy, antivirals such as Remdesivir and monoclonal antibodies such as Toclilizumab- an interleukin-6 receptor antagonist). The decision of delivery needs to balance the risk of fetal prematurity, concerns regarding fetal health, the trajectory of maternal respiratory status as well as maternal hemodynamic and inflammatory burden following major surgery such as caesarean section. Table 2 summarizes the current triaging system and management of COVID positive women at different stage of illness. The recommendations from the multidisciplinary meeting are shared with the woman and her partner during teleconsultation session.

**Table 2:** Escalation plan for management of pregnant woman with COVID-19 infection in our institution (adapted from RCOG guideline)

Clinical Category	Clinical criteria	Action plans in our institution
**Green**	• SpO_2_ 94–100% on RA and RR ≤ 20	• Ensure no obstetric/fetal or other medical concerns • Consider discharging when woman is out of acute phase of illness and low infectious status • COVID specific treatment ■ Consider role of monoclonal antibody (sotrovimab, casirivimab+imdevimab) or short course intravenous remdesivir for unvaccinated/seronegative womenearly in illness onset with co-morbidities who are deemed at high risk of progression to severe illness.
**Yellow**	• SpO2 94–100% on FiO2 ≥ 28%	• Assessment by multidisciplinary team ✓ Discuss timing of birth and delivery plans ✓ Assessment by infectious disease specialists • Depending on the gestational age ✓ Consider steroids for fetal lung maturity (if at risk of preterm delivery < 35 + 6 weeks) ✓ Consider magnesium sulfate for neuroprotection (if at risk of preterm delivery < 34 weeks) • COVID specific treatment ✓ Dexametasone +/− Remdesivir
**Amber**	• SpO2 94–100% on FiO2 ≥ 35%	• Assessment by multidisciplinary team ✓ Refer to ICU team ✓ Discuss the risk and benefits of emergency caesarean birth • Depending on the gestational age ✓ Consider steroids for fetal lung maturity (if at risk of preterm delivery < 35 + 6 weeks) ✓ Consider magnesium sulfate for neuroprotection (if at risk of preterm delivery < 34 weeks) • COVID specific treatment ✓ Dexametasone +/− Remdesivir ✓ Consider tocilizumab for women at high risk of or who are exhibiting rapid respiratory decompensation due to COVID-19 associated systemic hyperinflammation. ✓ Consider use of high flow oxygen ✓ Consider awake proning position when feasible
**Red**	• SpO2 < 94% on FiO2 ≥ 60%	• Assessment by multidisciplinary team ✓ Urgent review by ICU team and obstetric team ✓ Discuss timing of intubation ✓ Discuss risk and benefits of emergency caesarean birth for maternal resuscitation • Depending on the gestational age ✓ Consider steroids for fetal lung maturity (if at risk of preterm delivery < 35 + 6 weeks) ✓ Consider magnesium sulfate for neuroprotection (if at risk of preterm delivery < 34 weeks) • COVID specific treatment ✓ Consider early intubation ✓ Dexamethasone +/− Remdesivir ✓ Consider tocilizumab for women at high risk of or who are exhibiting rapid respiratory decompensation due to COVID-19 associated systemic hyperinflammation.

In our institution, intubation of COVID infected pregnant women is preferably performed by the anaesthesia team in view of the increased risk of difficult airway associated with pregnancy. Rapid desaturation and potential hemodynamic instability following the short apnea period resulting from both physiological changes in pregnancy and severe COVID pneumonia can be expected and plans for resuscitation should be conceived and all necessary staff, equipment and medication for resuscitation should be available before commencing intubation. We prefer to intubate and stabilize critical patients in the isolation room, before any planned transfer out to the operating theatre or to the ICU. In critical patients with extremely borderline respiratory status, we may even have obstetricians and neonatologists standby to perform a perimortem caesarean, if the patient becomes unstable after intubation. Otherwise, if there are plans to deliver the fetus after intubation, then the operating theatre staff would be informed to receive the patient for emergency caesarean section afterwards. A detailed summary of the pregnant women with COVID infection who had given birth in our institution can be found in Table 3.

**Table 3:** Summary of maternal characteristics and neonatal outcome of deliveries in women with COVID-19 infection in our institution

Patient Number	Maternal status				Neonatal status						
	Diagnosis	Day of illness	CT value (E/N2)	Vaccination status	Gestational age	Weight(g)	Mode of delivery	Resuscitation	Apgar score @ 1& 5 min	Neonatal admission location	COVID-19 PCR
	COVID- 19 URTI	17	24.1/26.4	No	37 + 6	2570	NVD	CPAP	8 & 9	NICU (Isolation)	Neg
	COVID- 19 URTI	3	25/26.8	No	39 + 3	3230	NVD	Nil	8 & 9	Nursery (Isolation)	Neg
	COVID-19 URTI	13	22.1/ 24.1	No	38 + 4	3230	NVD	Nil	8 & 9	Nursery (Isolation)	Neg
	Severe COVID-19 Pneumonia	10	29.2/30.8	No	29 + 4	1435	Emergency LSCS	Intubation	5 & 9	NICU (Isolation)	Neg
	COVID-19 URTI	3	13.7/14.2	No	39 + 4	3475	NVD	Nil	9 & 9	Nursery (Isolation)	Neg
	COVID-19 URTI	3	11.7/13.6	No	35 + 6	2295	Emergency LSCS	Nil	8 & 9	High Dependency (Isolation)	Neg
	COVID-19 URTI Thyroid toxicosis	13	20.4/22.7	No	35 + 3	2310	Emergency LSCS	CPAP	5 & 7	NICU (isolation)	Neg
	Asymptomatic GDM	1	31.3/34.7	Yes	36 + 5	2640	Emergency LSCS	Nil	8 & 9	Nursery (Isolation)	Neg
	Severe COVID-19 Pneumonia	8	15.67/15.64	Partial. D1 8 days prior to delivery	32 + 6	1810	Emergency LSCS	CPAP	4 & 7	NICU (Isolation)	Neg
	COVID-19 URTI	5	17.02/16.79	No	38 + 3	2790	NVD	CPAP (1 min)	7 & 8	NICU (High Dependency)	Neg

*GDM* Gestational Diabetes Mellitus, *URTI* Upper Respiratory Tract Infection

### Considerations in the provision of anaesthesia and analgesia for COVID positive woman

In general, the anaesthesia goals for taking care of COVID positive women include optimal labour analgesia and anaesthesia for the women while not compromising the safety of both the woman and the healthcare professionals. Neuraxial techniques have been recommended as the gold standard both for labour analgesia and anaesthesia for caesarean section.

Early labour epidural placement is also recommended in covid patients in international guidelines. Previous literature has also suggested that intrapartum cesarean section under general anaesthesia should be avoided whenever possible. A well-working epidural catheter will reduce the odds of general anaesthetic and the aerosol-generating intubation and extubation process associated with general anaesthetic. However, different concerns regarding labour epidural for this group of women have also been raised.

Contraindications for epidural such as thrombocytopenia has been reported in almost one third of COVID women [13–15]. Coagulopathy is often a worry as well as anticoagulation in the form of prophylactic LMWH is often given to prevent the hypercoagulation state from both pregnancy and underlying COVID infection [11, 16]. Moreover, technical issues due to the donning of personal protective equipment (PPE) pose further challenges to the proceduralists due to the alteration of procedural sensation and accuracy, increasing the difficulty and complications [13]. Some authors have also raised concerns about the increased incidence of peripartum fever after epidural insertion as well as theoretical risk of meningitis in a woman with active viral infection [17].

Other currently available alternative analgesia options for COVID positive women in labour include Entonox and patient controlled (PCA) remifentanil. There has been a continuous debate over the administration of Entonox to COVID positive women. Some believe that it may increase the risk of droplet generation and contaminate the breathing circuit, while some consider it not an aerosol-generating procedure [18, 19]. Very limited literature exists so far regarding the use of PCA remifentanil for this group of women, mainly limited to a few case reports [19]. The concern focuses on the potent respiratory suppression effect of remifentanil on women who are already having respiratory symptoms [20]. It is generally believed that remifentanil is safe for women with no active respiratory symptoms or abnormal saturation. However, close monitoring for COVID positive women is essential and critical.

In our institution, the choice of labour analgesia is decided on a case-by-case basis, taking into consideration the woman’s own wishes, active COVID disease status, previous obstetric history and likelihood of progressing into emergency cesarean section etc. [13]. Epidural performed under full PPE is recommended as the first line for labour analgesia for COVID women in labour unless contraindicated. Platelet count should be closely monitored in COVID positive women, as a rapidly down-trending platelet might be a concern [14]. Entonox has also been made available in the isolation ward using portable Entonox cylinders. PCA remifentanil use is strictly reserved for women contraindicated for epidural insertion but without respiratory symptoms, clinically stable, and with adequate nursing staff available to monitor the woman for respiratory suppression. Most importantly, a continuous discussion between the infectious disease specialists, obstetrician and anaesthetists taking care of such women need to be carried out at all times.

### Considerations in the postpartum care of COVID positive women and neonates

The postpartum period, also known as the “fourth trimester”, is a critical period for both a woman and her infant. Despite being nursed in isolation wards, the COVID-19 positive women are provided routine postnatal care to help them recover from childbirth and adapt to the physical, social and psychological changes that they may experience postnatally. All pregnant women who have been hospitalized and have had confirmed COVID-19 infection are offered thromboprophylaxis during their inpatient stay and for 10 days following hospital discharge.

#### Considerations for disposition of babies following birth

Rooming-in comes with its attendant benefits including greater satisfaction, bonding, and ease of establishment of breastfeeding [21]. WHO recommends early and uninterrupted skin-to-skin contact, rooming-in and kangaroo mother care, as these practices significantly improve neonatal survival and reduce morbidity [22, 23]. Current evidence suggests that rooming-in is generally safe, however this remains a controversial topic due to the reported 3.7% risk of horizontal transmission of the COVID-19 virus from the symptomatic mother to her baby, especially those with high viral loads [24]. Several earlier guidelines from various countries have recommended separation to minimize risk of horizontal transmission from mother to baby [25–28].

Case reports as well as a few observational studies have supported the practice of rooming-in due to low risk of horizontal transmission [29–33]. A prospective cohort study in the UK investigating SARS-CoV-2 infection in the first 28 days of life found that neonatal infection is uncommon and unlikely [30]. In a separate study on a limited number of neonates born to COVID-19 positive mothers from India, it was found that maternal viral load was not found to be associated with the positivity status or severity of the illness of neonate [31]. Studies from Italy and Turkey have similar findings as well [32, 33].

In addition, rooming-in option is potentially less resource intensive. With both mother and baby in a single room instead of two separate rooms, the utilization of isolation rooms, is reduced by 50%. This is particular important when a particular region is facing a large wave of COVID-19 infection in the community, as isolation rooms especially negative pressure rooms are scarce resources.

Lastly, there is suggestion from a single-arm cohort study in China that mother–baby separation is correlated with negative effects on developmental domains including communication, gross motor, and personal–social [34] (Table 4).

**Table 4:** Pros and Cons of room-in versus temporary separation

Neonatal Considerations	Pros	Cons
**Room-in**	✓ Potentially less resource intensive during the pandemic ✓ Enhance bonding between mother and baby and long-term neonatal development ✓ Promotes establishment of breastfeeding and inherent benefits of breastmilk, including presence of SARS-CoV-2 specific IgA and IgG in the milk	✓ Risk of horizontal transmission
**Temporary separation**	✓ Reduce risk of horizontal transmission ✓ Benefits of breast milk can still be reaped through feeding of Expressed Breast Milk (EBM) ✓ Symptomatic mothers who are unwell and physically unfit to take care of baby would benefit from help from hospital staff who temporarily take care of baby	✓ Limits establishment of mother-baby bonding

#### A fine balance - “rooming-in” when it is “safer”

A risk stratification approach is used. Such a risk stratification approach can be based on clinical and/or laboratory criteria. An example of an approach based on clinical criteria is the Communicable Disease Center (CDC) (52) guidance on rooming-in, where mothers are encouraged to room-in if they meet the following criteria: beyond 10 days of illness, afebrile for > 24 h and clinically well.

We use a risk-stratification approach based on the following considerations:

I The infectivity of the mother

In our institution, the infectivity of the mother is based on both clinical and laboratory criteria. A mother is deemed to have a “lower risk” of horizontal transmission when the cycle threshold (CT)-value is > 25, and she is more than one to 2 weeks into her illness. A mother deemed to be at “higher risk” of horizontal transmission when her CT value is < 25 or she is in the early stages of the disease. These criteria may change as our understanding of COVID-19 evolves.

II. Clinical status of mother

We take into consideration the overall well-being of the mother and her ability to take care of her baby.

After considering the above and counselling the woman and her partner, parental autonomy is respected and a shared decision is made on the disposition of the baby. We conduct the counselling by means of a multidisciplinary video-consultation session. Mothers who choose to room-in would need to observe certain precautions to minimize the risk of horizontal transmission. These include wearing a facemask, practicing strict hand hygiene and keeping a distance of > two-meter between mother and baby when not feeding. Parents who choose to temporarily separate from their babies may still provide expressed breast milk for their babies. In our situation where parents opted for temporary separation, we used teleconferencing via smart tablets to allow parents to see and speak to their babies and are given devices to view their babies remotely. Following our own approach developed, there has been no horizontal transmission observed from mother to baby in our institution (Table 3).

#### Considerations for breastfeeding in the context of COVID-19

Breastfeeding is the cornerstone of infant survival, nutrition and development. The World Health Organization (WHO) recommends exclusive breastfeeding for the first 6 months of life. However, there are theoretical concerns of horizontal transmission of the SARS-CoV-2 virus from symptomatic mothers to their newborns through breastfeeding leading to debate in this topic. The WHO recommends that mothers with suspected or confirmed COVID-19 should be encouraged to initiate or continue to breastfeed on the basis that the benefits of breastfeeding substantially outweigh the potential risks for transmission.

Studies have shown the breastmilk of COVID-19 positive mothers contain antibodies that are protective. Pace et al. found that 76% of the milk samples collected from women with COVID-19 contained SARS-CoV-2-specific IgA, and 80% had SARS-CoV-2-specific IgG [35]. In addition, 62% of the milk samples were able to neutralize SARS-CoV-2 infectivity in vitro. Breastmilk from mothers in Israel who have received the mRNA COVID-19 vaccination for more than 6 weeks previously have also been found to contain protective antibodies [36].

After reviewing the above evidence and guidelines, in our institution, we recommend direct breastfeeding when the mother chooses to room-in and provision of expressed breast milk for mothers who choose temporary separation. Where there is insufficient mother’s own milk, pasteurized donor human milk (PDHM) is offered. Our institution is a partner of the Temasek Foundation Milk Bank programme (‘Donor Human Milk Bank Programme’, n.d.). Under the programme, human milk donated by nursing mothers who are not biologically related to the recipient baby, is screened, processed and dispensed by prescription in accordance to the international guidelines and protocols from the UK National Institute of Health Care (NICE) and the Human Milk Banking Association of North America (HMBANA) [37].

#### Maternal and fetal outcomes of pregnant women with COVID-19 infection

At the time of manuscript writing, we have delivered ten pregnant women with COVID infection. All ten women were infected with COVID in the third trimester of their pregnancy, were monitored in our institution’s isolation maternity unit and were managed by the multidisciplinary team.

Out of the ten women with COVID infection, one was asymptomatic, seven had mild respiratory symptoms and two had severe pneumonia (one required high flow oxygen therapy and another required extracorporeal membrane oxygenation support). The woman who was asymptomatic for COVID was fully vaccinated against COVID while the remaining women were unvaccinated.

Out of those women with asymptomatic or mild respiratory symptoms, five of them had full term vaginal births, one had thyrotoxicosis which required caesarean section at 35 + 3 weeks gestation and two women went into preterm labour and underwent caesarean section for previous caesarean section (delivered at 35 + 6 weeks and 36 + 6 weeks gestation). The two women with severe pneumonia had caesarean section for maternal indications at 29 + 4 weeks and 32 + 6 weeks gestation. Postnatally, their clinical conditions improved and were all discharged well.

Our caesarean section rate for women with COVID infection is 50% and out of those who required caesarean section, 40% were for severe COVID pneumonia. Our outcomes are similar to those published in the literature. In the systemic review and meta-analysis conducted by *Jafari* et al, they reported that caesarean sections occurred in 48% of the pregnant COVID cases and among cases with available indications for caesarean sections, 55.9% was due to COVID pneumonia [38].

Fifty percent of babies were born preterm in our cohort, as compared to the national average of 10%, partially due to the maternal respiratory conditions [39]. Overall, the clinical course of the babies is on par with babies of the same degree of prematurity.

## Conclusion

In the current COVID-19 pandemic, a coordinated multidisciplinary care approach is critical in taking care of COVID-19 pregnant women who are at risk of COVID-19 related complications while maintaining the safety of the healthcare team. Simulation activities are important to improve the workflow process, refine the protocols and identify latent gaps in the preparation process. Patients’ autonomy should be respected through a shared decision-making process by providing them with the latest evidence on labour and postpartum care in the current pandemic.

## Data Availability

All data generated or analysed during this study are included in this published article.
